# Supplementary material for: Molecular characterization of Cdh12-SCON conditional knockout mice reveals unexpected splicing changes
Source: Transgenic Res. 2026 May 2;35(1):19. doi: 10.1007/s11248-026-00496-7 (PMC13135578; doi:10.1007/s11248-026-00496-7)
Supplement: Supplementary file 1 — Supplementary file1 (DOCX 813 kb) [file 11248_2026_496_MOESM1_ESM.docx]

**Table S1 | Oligonucleotides used for the generation and validation of the Cdh12^SCON^ allele**

| Oligo | Sequence |
| --- | --- |
| gRNA | CACTCTTCGTGCTCAGGCAG |
| ssDNA | TGCAATAAGAAGCCTGGATAGAGAAGAAAAACCTTTCTACACTCTTCGTGCTCAGGTAAGTAATAACTTCGTATAAGGTATCCTATACGAAGTTATTCTCTCTGCCTATTGGGGTTACAAGACAGGTTTAAGGAGACCAATAGAAACTGGGCATGTGGAGACAGAGAAGACTCTTGGGTTTCTGATAGGCACTGACATAACTTCGTATAAGGTATCCTATACGAAGTTATTTTCCCTCCCTCAGGCAGTGGACATAGAAACCAGGAAGCCACTGGAGCCTGAATCAGAGTTCATCATTAA |
| Int2/F | CTGAGGAGTTAAGGGTGCATTC |
| Int3/R | TGCAGTCTGTTGGTTAATAGCA |
| SCON/R | GTCTTGTAACCCCAATAGGCAGA |

**Table S2 | PCR assays with their corresponding primers, Tm and thermocycling program.**

| Assay | Forward primer | Reverse primer | Tm | Program^1^ |
| --- | --- | --- | --- | --- |
| SCON genotyping | GTTTCTCAGCTGCATTCGGAC | TGCAGTCTGTTGGTTAATAGCA | 60°C | 1 |
| CRE genotyping | GCCTGCATTACCGGTCGATGCAACGA | GTGGCAGATGGCGCGGCAACA CCATT | 67°C | 2 |
| Exon2-exon12 | TGGCTGGGTATGGAATCAGT | CGGCTTCTGTGGTGAGAGAG | 67°C | 3 |
| Exon2-SCON | AGCCCCAACAGACTTTAGCC | CTGAGGGAGGGAAAATAACTTCG | 62.2°C | 1 |
| Exon3a-SCON | TGGTGCTGGCACTGTTTTTAC | CTGAGGGAGGGAAAATAACTTCG | 62.2°C | 1 |
| SCON-exon3b | TACGAAGTTATTTTCCCTCCCTC | ACTAGCAACATAAGGTCCATCCA | 62.2°C | 1 |
| SCON-exon4 | TACGAAGTTATTTTCCCTCCCTC | CTTCACCTGCAGCACATACG | 62.2°C | 1 |
| SCON-exon5 | TACGAAGTTATTTTCCCTCCCTC | CCTCCCATGTCTTTCGCTTG | 62.2°C | 1 |
| SCON-exon6 | TACGAAGTTATTTTCCCTCCCTC | ACAGGGGAAGACTCGGGAAC | 62.2°C | 1 |
| SCON-exon7 | TACGAAGTTATTTTCCCTCCCTC | GGTTTGCTGAACACTGGTGG | 62.2°C | 1 |
| Exon2-exon3a | AGCCCCAACAGACTTTAGCC | AGCACGAAGAGTGTAGAAAGGT | 67.5°C | 1 |
| Exon2-exon4 | AGCCCCAACAGACTTTAGCC | CTTCACCTGCAGCACATACG | 67.5°C | 1 |
| Exon2-exon5 | AGCCCCAACAGACTTTAGCC | CCTCCCATGTCTTTCGCTTG | 62.2°C | 1 |
| Exon2-exon6 | AGCCCCAACAGACTTTAGCC | ACAGGGGAAGACTCGGGAAC | 70°C | 1 |
| Exon2-exon7 | AGCCCCAACAGACTTTAGCC | GGTTTGCTGAACACTGGTGG | 70°C | 1 |
| Exon3a-exon4 | TGGTGCTGGCACTGTTTTTAC | CTTCACCTGCAGCACATACG | 70°C | 1 |
| Exon3b-exon4 | GGACATAGAAACCAGGAAGCCA | CTTCACCTGCAGCACATACG | 70°C | 1 |

^1^Program 1 corresponds to 03:00 (95°C); 35 x 00:30 (95°C), 00:30 (Tm), 01:00 (72°C); 05:00 (72°C), program 2 to 05:00 (95°C); 35 x 00:30 (95°C), 00:30 (Tm), 01:00 (72°C); 10:00 (72°C), and program 3 to 03:00 (98°C); 35 x 00:10 (98°C), 00:30 (Tm), 01:20 (72°C); 05:00 (72°C).

**gBlock sequence**

5’CACCACTTCAGCCACAGCCCCAACAGACTTTAGCCACAGAACCAAAAGAAAATGTTATCCACCTTTCGGGGAGACGATCCCATTTCCAACGAGTTAAACGTGGCTGGGTATGGAATCAGTTTTTTGTGCTGGAAGAGTACATGGGCTCCGAACCTCAATATGTGGGGAAGCTGCATTCGGACTTGGATAAAGGAGAGGGCACTGTTAAATACACGCTCTCCGGAGATGGTGCTGGCACTGTTTTTACAATTGATGAAACTACAGGAGACATTCATGCAATAAGAAGCCTGGATAGAGAAGAAAAACCTTTCTACACTCTTCGTGCTCAGGTAAGTAATAACTTCGTATAAGGTATCCTATACGAAGTTATTTTCCCTCCCTCAGGCAGTGGACATAGAAACCAGGAAGCCACTGGAGCCTGAATCAGAGTTCATCATTAAAGTGCAGGATATTAATGACAATGAACCAAAGTTTTTGGATGGACCTTATGTTGCTAGTGTTCCAGAAATGTCTCCTGTGGGTGCGTATGTGCTGCAGGTGAAGGCCACAGATGCAGACGATCCTACCTATGGGAACAGTGCCAGAGTTGTTTACAGCATCCTTCAGGGGCAACCTTATTTCTCTATTGATCCCAAAACAGGTGTCATTAGAACAGCATTGCCAAACATGGACAGAGAAGTCAAAGAGCAGTACCAGGTCCTCATTCAAGCGAAAGACATGGGAGGACAGCTCGGAGGACTTGCTGGAACTACAGTTGTCAACATCACCCTTACTGATGTCAATGACAACCCACCTCGCTTCCCAAAAAGCATCTTCCATCTGAAAGTTCCCGAGTCTTCCCCTGTTGGCTCAGCTATTGGAAGAATAAGAGCAGTAGATCCTGATTTTGGAAAAAATGCAGAAATTGAATACAACATTGTCCCAGGAGATGGGGGAAATTTGTTTGACATTGTCACAGATGAGGATACACAAGAAGGAATCATCAAATTGAAAAAGCCTTTAGATTTTGAAACCAAGAAGGCATATACTTTTAAAGTAGAGGCCTCCAACCTTCACCTTGACCACCGCTTTCACTCTGCTGGGCCATTTAAGGATACTGCTACAGTAAAAATCAGCGTGCTGGATGTGGATGAGCCACCAGTGTTCAGCAAACCACTGTACACCATGGAGGTTTATGAAGACACTCCTGTGGGGACCATCATCGGAGCTGTCACAGCACAAGACCTTGATGTGGGCAGTAGTGCTGTTAG 3’


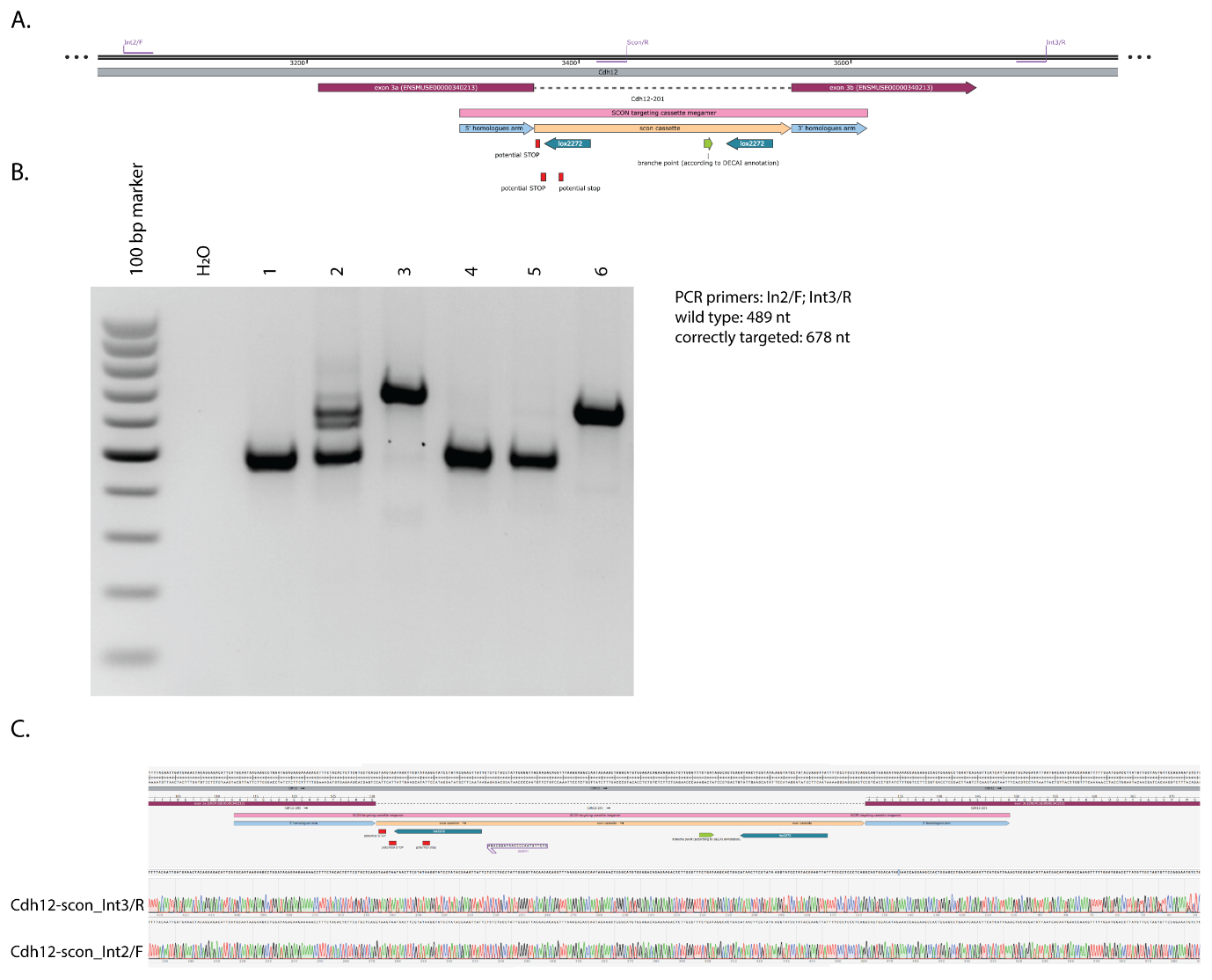
**Figure S1. Generation and quality control Cdh12^SCON^ model. A.** Predicted and annotated Cdh12-scon locus. **B.** Pre-screen PCR of Cdh12^SCON^ founder animals. The higher bands from animals 2, 3 and 6 were isolated from gel and sequenced. Animal 3 was found to be correct and was used for subsequent colony building. **C.** Sequence verification of Cdh12^SCON^ allele after gel purification. The figure was compiled from multiple screen shots using Photoshop for representative purposes. The indicated forward and reverse Sanger sequence traces are included as Supplementary files 2 and .3


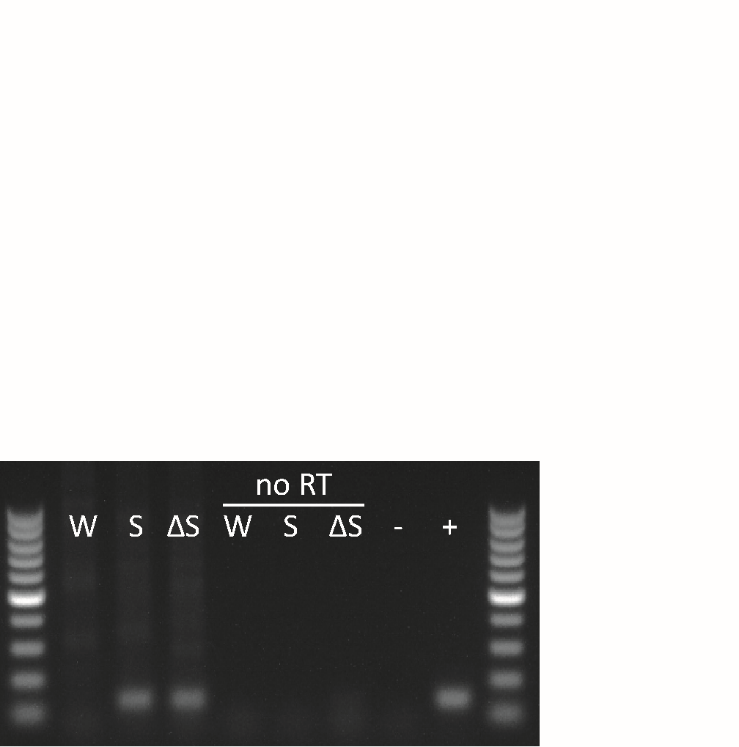


**Figure S2. gDNA contamination test for (Δ)SCON – exon 3b.** PCR using the (Δ)SCON – exon 3b assay on RNA with and without reverse transcriptase (RT). Marker is 100 bp ladder. W = wildtype, S = Cdh12^SCON/SCON^, ΔS = Cdh12^ΔSCON/ΔSCON^, - = MQ, + = gBlock.
